# Geographical and Temporal Variability of Ultra-Processed Food Consumption in the Spanish Population: Findings from the DRECE Study

**DOI:** 10.3390/nu14153223

**Published:** 2022-08-06

**Authors:** Carmen Romero Ferreiro, Pilar Cancelas Navia, David Lora Pablos, Agustín Gómez de la Cámara

**Affiliations:** 1 Scientific Support Unit, Instituto de Investigación Sanitaria Hospital Universitario 12 de Octubre (imas12), 28041 Madrid, Spain; 2 Spanish Clinical Research Network (SCReN), 28040 Madrid, Spain; 3 Faculty of Health Sciences, Universidad Francisco de Vitoria, Pozuelo de Alarcón, 28223 Madrid, Spain; 4 Consorcio de Investigación Biomédica en Red de epidemiología y salud pública (CIBEResp), 28029 Madrid, Spain; 5 Faculty of Statistical Studies, Universidad Complutense de Madrid (UCM), 28040 Madrid, Spain

**Keywords:** ultra-processed foods, NOVA classification, geographic variability, dietary patterns

## Abstract

The consumption of ultra-processed foods (UPFs) has increased in recent decades, worldwide. Evidence on the negative impacts of food processing on health outcomes has also been steadily increasing. The aim of this study is to describe changes in consumption patterns of ultra-processed foods in the Spanish population over time and their geographical variability. Data from four representative cohorts of the Spanish population were used (1991–1996–2004–2008). Dietary information was collected using a validated frequency questionnaire and categorized using the NOVA classification. A total increase of 10.8% in UPF consumption between 1991 and 2008 was found in Spain (*p*-value < 0.001). The products contributing most to UPF consumption were sugar-sweetened beverages, processed meats, dairy products, and sweets. Those who consumed more ultra-processed foods were younger (*p*-value < 0.001) and female (*p*-value = 0.01). Significant differences between the different geographical areas of Spain were found. The eastern part of Spain was the area with the lowest UPF consumption, whereas the north-western part was the area with the highest increase in UPF consumption. Given the negative effect that the consumption of ultra-processed foods has on health, it is necessary to implement public health policies to curb this increase in UPF consumption.

## 1. Introduction

Non-communicable diseases (NCDs) are the leading causes of disability and death worldwide and currently account for more than half of the global burden of disease [1,2]. One of the main public health objectives is to prevent and combat the development of the most prevalent non-communicable chronic diseases (cardiovascular disease, diabetes, obesity, high blood pressure, chronic respiratory disease, and some types of cancer), which are largely the result of excessive or unbalanced consumption of certain foods and/or nutrients [3,4], among other factors. Conventional teaching and practice on nutrition and health usually focuses on nutrients, or else on specific foods and drinks [5]. However, the issue of food processing is largely ignored or minimized in food and nutrition, and also in public health policies. It is now acknowledged that some of these chronic diseases have as one of their major causes increased consumption of ultra-processed foods [6,7,8].

Ultra-processed foods (UPF) are industrial formulations performed from substances derived from food or synthesized in laboratories (dyes, flavorings, and other additives). These foods generally contain little or no natural foods, have also high amounts of fat, salt, or sugar, and low fiber, protein and micronutrients content [9,10]. They are distinguished as food products of low nutritional quality [11,12,13,14,15]. In this group, a large variety of industrially processed food products, such as some pastries, savory snacks, reconstituted meat products, pre-prepared frozen dishes, and soft drinks, among other food items, are included.

Evidence on the relationships between food processing and health outcomes has been increasing steadily in the last years. UPFs are prevalent in diets worldwide, contributing from 20% to more than 60% of total energy intake, depending on the country and age range [16,17,18]. UPFs account for more than 50% of total daily energy consumption in some high-income countries, such as the United States [19], the United Kingdom [20], Australia [21], and Canada [22]. The consumption of UPF has been associated with unhealthy dietary patterns [11,12,13,15,23,24,25,26,27,28] and with overweight and obesity in studies conducted in the United States [29], Canada [30], France [31], Brazil [32,33], and in most Latin American [34,35] and European [36] countries. Other recent cohort studies from Spain and France found relationships between UPF and hypertension [37,38] and cancer [39], respectively. In addition, some studies reported results on the negative effect of ultra-processed food consumption on all-cause mortality [40,41,42,43,44].

Globally, between 1990 and 2010, the consumption of unhealthy food items worsened, with heterogeneity across regions and countries [45]. Among unhealthy foods, consumption of ultra-processed foods is on the rise [8,34,46] around the world. In Spain, the percentage of ultra-processed foods of all food purchases almost tripled between 1990 and 2010 (from 11.0% to 31.7%) [47]. In addition, the burden of chronic non-communicable diseases also increased by approximately 4% between 1990 and 2010 in Spain [48,49], and is estimated to increase further in the forthcoming years. Several studies report that consumption of ultra-processed foods in Spain accounts for approximately 24.4% of total energy intake [43,44], but these studies calculate consumption at a given point in time. There are no previous reports on the evolution of ultra-processed consumption over time (just about purchases) and its geographical distribution in Spain. In this context of the growing trends in chronic diseases, it is important to know the pattern of consumption of these products over time in order to understand the connection between diet and public health. In addition, factors such as cultural differences, education, personal tastes and traditions, geographic location, access to technology, and health and health attitudes are known to influence food availability and food preferences [50], so it is of particular interest to study the geographical distribution of food consumption.

The aim of the study was to describe changes in the consumption pattern of ultra-processed foods in the Spanish population over time (1991–1996–2004–2008), according to eight geographical regions.

## 2. Materials and Methods

### 2.1. Design and Participants

The multicentre population-based study Diet and Risk of Cardiovascular Disease in Spain (DRECE) was used as a substrate for analysis. DRECE [51] was designed in 1991 to determine the real situation of the Spanish population with regard to the risk of cardiovascular disease (CVD), based on the prevalences of risk factors and their relationships with dietary habits. DRECE I (1991) was a representative sample of the Spanish population stratified by age, sex, and geographical areas. After 5 and 12 years, DRECE II (1996) and DRECE III (2004), two subgroups of the original DRECE cohort, were undertaken. Nearly 20 years after the start of DRECE, the capacity to locate and re-screen cohort participants for follow-up was reduced and biased to scientifically unprofitable extremes. For this reason, in 2008 the DRECE Institute for Biomedical Studies formulated a new breakthrough strategy and undertook the DRECE IV study. To this end, a new cohort was recruited, with respect to the initial distribution in eight geographical regions and the same conditions of DRECE I to make it a representative sample of the current Spanish population and an extension of the DRECE project. This study will compare the above mentioned DRECE cohorts. DRECE I (1991) consists of 4787 persons, DRECE II (1996) consists of 1079 persons, DRECE III (2004) consists of 2009 persons, and DRECE IV (2008) consists of 5038 subjects with the same geographical and population strata design as the initial population. All cohorts have answered a food frequency questionnaire, designed and validated for epidemiological studies in the Spanish population [52,53].

### 2.2. Geographical Areas

**Figure 1:**
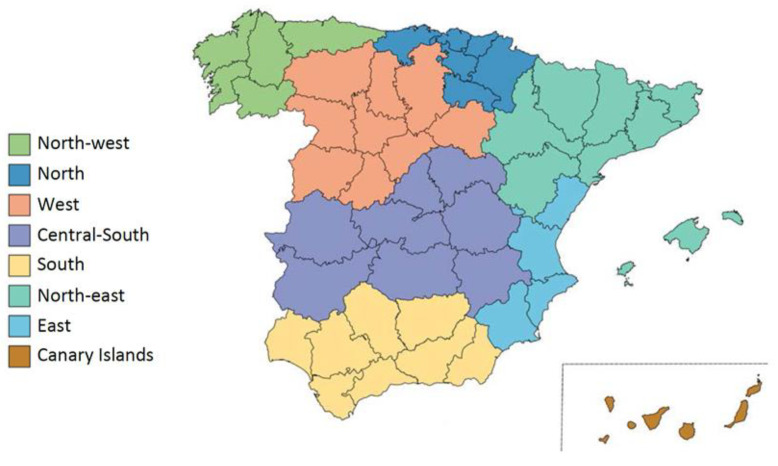
Geographical distribution of Spain in eight areas according to the Ministry of Agriculture, Fisheries, and Food (MAPA).

The geographical distribution was structured according to the area scheme of the food consumption panel of the Ministry of Agriculture, Fisheries, and Food (MAPA, acronym in Spanish) [54], previously described in Gómez Jerique et al. [51], and included the Canary Islands, north-east, Levante (East), Andalucía (South), central-south, Castilla y León (west), north-west, and north areas (Figure 1).

### 2.3. Dietary Assessment

The estimation of ultra-processed food consumption was carried out through the data collected in the dietary questionnaires. The first step in modelling dietary changes was to classify all foods according to the NOVA classification, developed in Brazil and used internationally in research [10,55]. The NOVA classification divides foods into four groups according to their degrees of processing: Group 1, unprocessed/minimally processed foods; Group 2, processed culinary ingredients; Group 3, processed products; Group 4, all ultra-processed foods. The full list of the recorded foods in the food frequency questionnaire and their NOVA classification is shown in supplemental Table S1. The kcal/day consumed from ultra-processed foods and their percentages of total kcal were then determined. Respondents with extreme total energy intakes (<200 kcal and > 5000 kcal) were excluded from the analysis [15]. Those with an extremely low BMIs (BMI < 13) were also excluded.

### 2.4. Statistical Analysis

All statistical analyses were performed using SAS© software (SAS Institute Inc., Cary, NC, USA), version 9.4 of the SAS System for Windows. Descriptive data are presented as mean and standard deviation (SD) for continuous variables, and categorical variables are expressed as absolute or relative frequencies. Food consumption according to the NOVA classification in the different cohorts globally and by geographical area was described by simple correspondence analysis. A ternary diagram represents this relationship [56,57]. A ternary diagram is a triangular graph that visualizes in a two-dimensional way the relationships between cohorts (represented by dots in the diagram) and the percentage of food consumption according to the NOVA classification (represented on each of the three axes). The study of the change in UPF consumption over time (between the four different cohorts) was carried out using a multivariate mixed model adjusted for age, sex, body mass index (BMI), and total energy intake. An unstructured covariance matrix was used. The intercept was considered a random effect, and the rest of the variables were used as fixed effects [58]. Comparisons between geographical areas were estimated using the chi-square test or Fisher’s exact test for categorical variables, and for continuous variables were estimated using ANOVAs. In each cohort, the consumption of ultra-processed foods is represented by density maps according to the eight geographical areas. *p*-values  < 0.05 were considered statistically significant.

## 3. Results

The final sample size included 4679 individuals in DRECE I, 928 individuals in DRECE II, 1065 individuals in DRECE III, and 4835 individuals in DRECE IV. The demographic characteristics of the four cohorts are shown in supplemental Table S2. Between 1991 and 2008, there was a general increase in total energy intake (kcal/day) in the Spanish population (Table 1). Average consumption of ultra-processed foods (NOVA group 4) was found to be 24.44% of the total energy intake in 1991 (DRECE I), 25.61% in 1996 (DRECE II), 27.48% in 2004 (DRECE III), and 31.09% in 2008 (DRECE IV) (Table 1). UPF consumption changed over time also in both sexes, from 24.48% in males and 24.39% in females in 1991, to 31.03% and 31.39%, respectively, in 2008. In addition, the same evolution was observed according to age group and BMI (Table 1).

**Figure 2:**
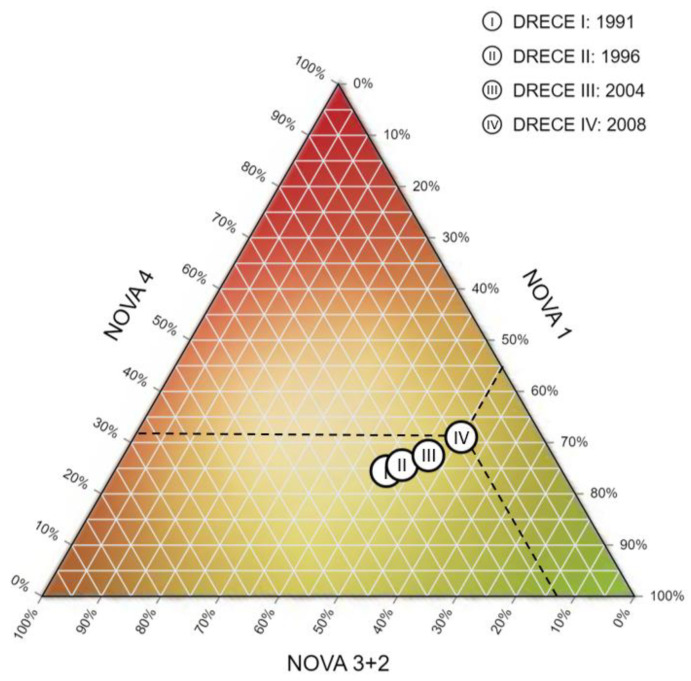
Ternary diagram of the average percentage of energy intake from the NOVA classification by the Spanish population over time.

The mixed model shows a significant upward trend (all adjusted *p*-values <0.001) in the consumption of ultra-processed products over the 17 years of the study, and a 10.79% ± 0.39 increase in the consumption of this type of product in Spain between 1991 and 2008 (Table 2). This increase over time can be seen in the ternary diagram (Figure 2). In the ternary diagram, for better representation, the NOVA 2 and NOVA 3 groups are shown together, as NOVA 2 represents a very low percentage of consumption, and it was decided to unify processed culinary ingredients (NOVA 2) and processed foods (NOVA 3) into one category. The axes of the diagram correspond to the percentages of foods belonging to NOVA 1, NOVA 3+2, and NOVA 4 (these percentages are also shown in Table 1). The points represented in the diagram correspond to the four cohorts (1991, 1996, 2004, and 2008) according to the amounts of products they included from each of the different NOVA groups. As an example of an interpretation, using the 2008 cohort (DRECE IV), represented with dashed lines in Figure 2, 31.09% of the food consumed corresponded to ultra-processed foods (NOVA 4), 13.70% to processed foods (NOVA 3+2), and 55.21% to unprocessed or minimally processed foods (NOVA 1). This interpretation can be made in the same way for the rest of the points in the diagram.

As a result of the mixed model, it was also found that participants who consumed the most UPF had significantly higher intakes of total energy (β = 1.86, *p*-value < 0.001) and were mostly female (β = 1.06, *p*-value = 0.01) (Table 2). In addition, individuals who consumed more ultra-processed foods were younger (β = −0.15, *p*-value < 0.001). UPF consumption in young people remained above 30% at all time points (Table 1). No association was found between UPF consumption and BMI (β = −0.05, *p*-value = 0.19) (Table 2).

The main food groups contributing to ultra-processed food intake (>10% energy contribution) were sugar-sweetened beverages (i.e., soft drinks) (18.41%), milkshakes and juice boxes (17.53%), meat and meat products (16.38%), and dairy products (13.50%) in 1991; dairy products (i.e., yogurts, ice cream, or Petit Suisse) (17.51%), meat and meat products (15.06%), and sweets and cookies (11.79%) in 1996; meat and meat products (17.92%), dairy products (14.01%), and sugar sweetened beverages (13.64%) in 2004; and industrial cakes and pastries (19.69%), dairy products (17.41%), and sugar sweetened beverages (11.73%) in 2008.

**Figure 3:**
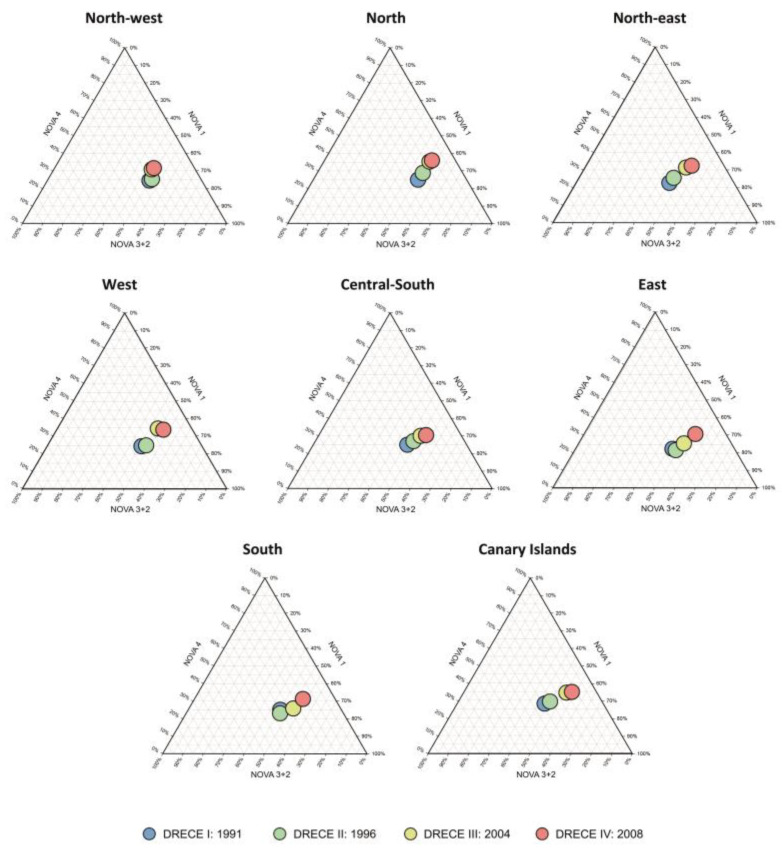
Ternary diagram of the average percentage of energy intake in the NOVA classification over time by geographical area of Spain.

The geographical study shows that in all cohorts the sample was homogeneous in terms of age and sex across the eight geographical areas (all *p*-values > 0.05) (Table 3). Significant differences in BMI, total energy intake, and ultra-processed food consumption were found between geographical areas at all time points (Table 3). When studying the consumption of ultra-processed foods by geographical area, the same trend was observed in all of them as in Spain as a whole: an increase over time in the consumption of this type of product (Figure 3). During the 17 years of the study, there was an overall increase in the consumption of ultra-processed foods of 11% in the north-west and north regions, 10.10% in the north-east, 9.41% in the west, 8.38% in the east, 6.70% in the Canary Islands, 6.13% in the south, and 5.20% in the central-south region.

**Figure 4:**
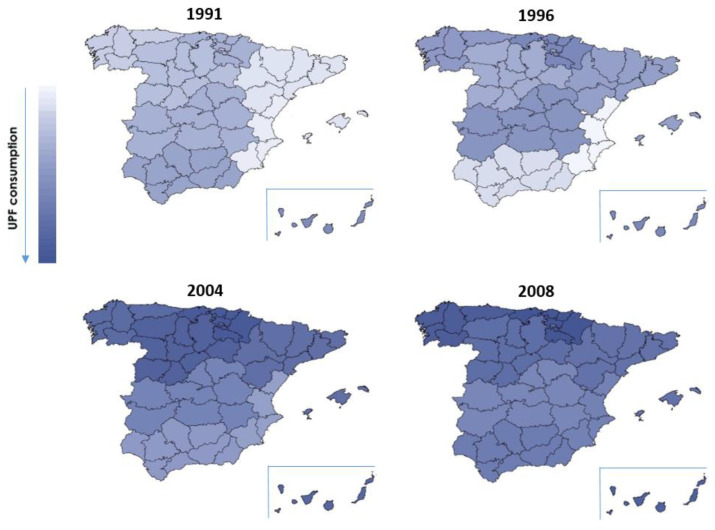
Geographical distribution of ultra-processed food consumption in Spain over time.

In 1991, the region with the highest consumption of ultra-processed foods was the Canary Islands, and in 2008 it was the northern region. As can be seen in Figure 4, the region with the lowest consumption of ultra-processed foods was the east, which was the region with the lowest consumption in 1991 (22.64%), 1996 (21.85%), and 2004 (25.75%), and had the second lowest in 2008 (31.03%). The Canary Islands was the region with the highest consumption of ultra-processed foods in 1991 (28.10%) and 1996 (29.33%), and then the northern region was the region with the highest consumption of ultra-processed foods in 2004 (35.34%) and 2008 (36.03%). The central-south region went from having intermediate consumption in the early years to becoming the region with the lowest consumption of ultra-processed foods in 2008, at 30.17%. The southern region started as one of the regions with the highest consumption of ultra-processed foods in 1991, and ended up as one of the regions with lower consumption compared to the rest. The western and north-western regions started with intermediate consumption but were among the regions with the highest consumption in 2004 and 2008, respectively. The north-east region retained intermediate consumption values compared to the rest of the regions consistently (Figure 4).

## 4. Discussion

About one third of daily energy intake was found to be provided by ultra-processed foods (UPF) in the Spanish population. Estimates of UPF purchases calculated from national household budget surveys (conducted in Europe between 1991 and 2008) showed that the average household availability of UPF ranged from 10% of total purchased dietary energy in Portugal to 50% in the UK [36]. In Spain, UPFs were found to contribute about 24–31% to total dietary energy (between 1991 and 2008), which is slightly higher than the average usual proportion of daily energy intake from UPFs (26.4%) found in this study. However, food consumption surveys often provide more details on the foods consumed compared to household budget surveys, which are based on purchases. When looking at published consumption data rather than household budget survey data, Spain is shown to be a country with a low consumption of ultra-processed foods compared to other countries, such as Canada (48%) [11], the United States (57.9%) [19], the United Kingdom (56.8%) [20], Belgium (about 33%) [28], and France (35.9%) [31]. These differences may be due to the fact that the data published in other countries correspond to different periods of time. They also could be due to the Mediterranean diet, which is characterized by high consumption of plant-based foods and fresh fruits, low consumption of red meat and other processed foods, the use of olive oil as the main source of fat, and a moderate intake of wine during meals [59]. In addition, other Mediterranean countries, such as Italy, also have lower UPF consumption (18%) [60].

On the other hand, a negative shift in the pattern of food consumption was found. UPF consumption has increased over time across the country. An increase of 10.79% in UPF consumption was found between 1991 and 2008 in Spain, from 1 in 4 foods being ultra-processed in 1991 to 1 in 3 in 2008, which is in line with the previously reported increase in UPF purchases between 1990 and 2010 in Spanish households [47]. As the nutrition literature increasingly recognizes ultra-processed foods (UPF) to be unhealthy, the diet in Spain can be considered increasingly unhealthy. This supports the evidence that between 1990 and 2010, diets based on unhealthy items worsened worldwide [45]. This trend has also been shown in other countries, such as Belgium [28], Sweden [61], the United Kingdom [20], and the United States [62]. This increase also parallels the growing burden in Spain and worldwide of non-communicable diseases [48,49], of which excessive consumption of ultra-processed foods is known to be one of the main causes [8,63]. The exact reasons for this increase in UPF consumption are not known, but may include the increased availability and accessibility of such products, as they are highly palatable and inexpensive, increased consumption of prepared foods outside the home over the past few decades, and aggressive and unregulated advertising of convenience foods, which may promote overconsumption [46,64]. The main groups of UPFs consumed in Spain were sugar-sweetened beverages; processed meats; dairy products; and sweets, biscuits, and cakes. These data are in line with those provided by the European household budget surveys (conducted between 1991 and 2008), where the most purchased UPFs were packaged breads, cakes, sweets and cookies, meat products, and sugar-sweetened beverages [36]. This also agrees with the most consumed UPFs in the United Kingdom, Belgium, Canada, and the United States [20,28,65]. It is worth noting that the consumption of processed meats decreased between 1991 and 2008 in Spain, from 16.38% to less than 10%, and the consumption of sugar-sweetened beverages from 18.41% to 11.73%. On the other hand, consumption of processed dairy products increased from 13.50% to 17.41%, and consumption of sweets from less than 10% to 19.69%. Similar results were found in young people in the United States between 1999 and 2018, where there was also a decrease in the consumption of sugar-sweetened beverages and an increase in the consumption of sweets [62]; and also in Sweden where there was a slight decrease in consumption of sugar-sweetened beverages between 2002 and 2010 [61]. This highlights the types of ultra-processed products for which there is most need to reduce consumption in the population and to implement policies to reduce their sales. Some countries, such as Uruguay [66] and Brazil [67], already include the concept of UPFs in food guidelines; and other countries, such as Mexico [68] and Hungary [69], have taken actions to limit the marketing of UPFs through taxation. Such policies do not exist in Spain and should start to be implemented in view of the evidence of the growing consumption of UPFs.

Young people consume the highest proportion of ultra-processed foods in their diets in the Spanish population, consistently—above 30%. Other studies, such as those from Belgium [28], the United States [70], Canada [11], Colombia [71], and Chile [26], have also found that children consume the highest amounts of UPF compared to other age groups. Given that young people are the highest consumers of UPFs, it could be beneficial to implement health policies targeting this population stratum in order to raise awareness of healthy food consumption. Higher UPF consumption was associated with higher BMI in other studies [29,30,32,36,61,72,73], but no such association was detected in Spain. Females consumed more UPF than males; this may be influenced by gender differences in food choices. Females appear to exhibit more stress-related eating behaviors [74], which may lead to higher UPF consumption.

Consumption of ultra-processed foods is high in all regions of Spain (21–36%). It is notorious that factors such as palatability and the high commercialization of these foods contribute to their presence in the eating habits of all families [75]. In addition, all regions saw a progressive increase in the consumption of this type of food (5.2–11%) during the 17 years of the study, similar to the overall increase in Spain. The Canary Islands is one of the regions with higher relative consumption of ultra-processed foods, which is in agreement with the dietary pattern found in other studies on this region, in which it has been characterized by high intakes of fats and carbohydrates (present at high levels in UPFs) with respect to other regions of Spain [76]. The north, north-west, and west regions showed worsening in their dietary patterns, being the regions with the highest increases in UPF consumption over time, and reaching the highest percentages of intake in 2008 (36%, 35.5%, and 34% of total intake, respectively) together with the Canary Islands. This may be due to the high carbohydrate and high fat consumption patterns of these regions, whose citizens have also been reported to have high HDL lipid profiles [76]. The eastern region remained over time one of the regions with the lowest consumption of UPFs, probably because it is geographically located on the Mediterranean coast and may be more deeply linked to the culture and traditions of a quality Mediterranean diet [77]. This has been evidenced by recent studies finding an inverse association between UPF consumption and adherence to the Mediterranean diet [78]. The north-east region retained average consumption over time, probably also due to its adherence to the Mediterranean diet because of its geographical position. Particularly, the southern and north-central regions are characterized by improved consumption patterns compared to the rest of the regions, being the regions with the lowest increases in UPF consumption over time. This is reflected in the micronutrient patterns of these regions, where low carbohydrate and protein intake and a low HDL lipid profile are reported [76]. The geographical variability found in UPF consumption in Spain has some consistency with the economic data provided by the National Statistics Institute (INE) [79]. The regions with the highest consumption of UPF in 2008 were those with the lowest growth in per capita household income in the 2000s. Along the same lines, the southern and central areas had the highest growth in per capita household income and the lowest growth in UPF consumption.

All these results reinforce the increase in the consumption of ultra-processed foods over the last few decades and the need for health policies that take into account the degree of food processing to address the increasing intake of UPFs.

There are several strengths to this study. The use of a large, nationally representative sample of the Spanish population maximizes generalizability. The testing of the same hypothesis both cross-sectionally and over time lends credibility to our results. Self-reported dietary intake data are less biased than purchasing data, as all meals consumed are included, including those consumed away from home, which are more likely to be ultra-processed. However, the study also has some limitations. Although the NOVA classification has been questioned sometimes, it is simple and clear to apply; no better alternative has yet been proposed. The food frequency questionnaire was not designed to collect data on consumption of UPFs according to the NOVA classification. Each food item was classified into its most likely NOVA group, but we cannot rule out misclassification of some foods. Finally, to minimize information bias, validated procedures were used, and subjects with inconsistent intake data were excluded. Finally, future studies in this field of research could consider including more qualitative data.

## 5. Conclusions

There has been an increase in UPF consumption over time in Spain, namely, of approximately 10.8% between 1991 and 2008. About 21–36% of the average daily energy intake is provided by UPFs, with differences depending on the geographical area. The products contributing most to UPF consumption are sugar-sweetened beverages, processed meats, dairy products, and sweets. Young people and females have the highest intakes of ultra-processed foods. No correlation was found between UPF consumption and BMI. The eastern part of Spain is the area with the lowest UPF consumption, and the north-western part of Spain is the area with the highest increase in UPF consumption. Given the robust scientific evidence associating UPF consumption with various adverse health outcomes, realistic public health policies are needed to limit the availability, affordability, and marketing of UPFs. In addition, raising awareness through educational programs that promote healthier food environments to individuals of all socio-demographic and socio-economic categories, but especially to the youngest, would be useful to prevent further increases in UPF consumption in Spain.

## Figures and Tables

**Table 1:** Food intake according to the NOVA classification over time (DRECE cohorts) and distribution of ultra-processed food consumption (NOVA 4) by sex, age, and BMI class.

	DRECE I 1991	DRECE II 1996	DRECE III 2004	DRECE IV 2008
**Total energy intake (kcal/day)**	2024.80 (727.09)	2362.49 (1197)	2373.91 (1068)	2441.01 (948.75)
**NOVA classification (% of energy)**				
NOVA 1	45.91 (13.33)	47.96 (15.58)	51.47 (14.01)	55.21 (12.13)
NOVA 3+2	29.65 (13.24)	26.43 (15.96)	21.05 (16.48)	13.70 (15.37)
NOVA 4	24.44 (13.95)	25.61 (16.29)	27.48 (19.17)	31.09 (19.24)
**UPF consumption (NOVA 4) (% of energy)**				
**By sex**				
Male	24.48 (13.89)	23.71 (16.76)	26.14 (19.72)	31.03 (17.57)
Female	24.39 (14.01)	27.83 (15.16)	29.01 (15.18)	31.39 (18.47)
**By age group**				
5–24	32.79 (12.83)	31.69 (14.84)	33.72 (14.04)	34.12 (11.48)
25–49	19.81 (11.62)	24.70 (16.63)	26.70 (17.26)	27.91 (21.01)
50–75	16.13 (11.41)	19.39 (19.01)	22.01 (12.29)	25.14 (19.83)
**By BMI class**				
Normal weight	16.96 (11.41)	23.67 (17.30)	22.76 (18.74)	27.93 (19.22)
Overweight	19.99 (12.45)	26.04 (16.15)	25.88 (19.86)	31.77 (18.90)
Obese	28.27 (13.85)	26.11 (15.06)	28.67 (18.67)	33.31 (21.15)

Data is shown as mean (SD).

**Table 2:** Mixed model coefficients for UPF consumption over time adjusted for age, sex, BMI, and total energy intake.

	Estimate	Standard Error	*p*
**Intercept**	24.49	1.21	<0.001
**Time (Cohorts)**			
DRECE I 1991	Ref.		
DRECE II 1996	5.31	0.62	<0.001
DRECE III 2004	9.63	0.66	<0.001
DRECE IV 2008	10.79	0.39	<0.001
**Age (years)**	−0.15	0.01	<0.001
**Sex**			
Male	Ref.		
Female	1.06	0.33	0.01
**BMI (kg/m^2^)**	−0.05	0.04	0.19
**Total energy intake (kcal/day)**	1.86	0.19	<0.001

AIC:3156  8154 subjects included.

**Table 3:** Ultra-processed food (NOVA 4) intake and demographic characteristic by geographical area.

	Geographical Areas								*p*
**DRECE I** **1991**	**North-West**	**North**	**North-East**	**West**	**Central-South**	**East**	**South**	**Canary Islands**	
***n***	514	422	683	341	913	553	1075	178	
**Age (years)**	30.04 (15.61)	30.38 (15.59)	31.74 (15.59)	30.22 (15.50)	30.54 (15.73)	31.57 (15.58)	29.89 (15.50)	29.48 (16.04)	0.175
**Sex (male)**	249 (48.44%)	206 (48.82%)	331 (48.46%)	171 (50.15%)	440 (48.19%)	263 (47.56%)	539 (50.14%)	89 (50.00%)	0.977
**BMI (kg/m^2^)**	24.43 (4.61)	23.53 (4.48)	24.43 (4.83)	23.65 (4.47)	24.05 (5.10)	24.14 (4.73)	24.75 (5.68)	24.09 (5.63)	0.002
**Total energy intake (kcal/day)**	1996.47 (641.84)	1942.73 (593.48)	2037.55 (707.23)	2152.04 (686.01)	2023.84 (693.92)	1964.44 (802.26)	2040.11 (796.11)	2109.13 (846.20)	<0.001
**NOVA classification**									
**GROUP 4 (% of energy)**	24.47 (14.45)	25.03 (12.95)	22.65 (13.75)	24.60 (12.94)	24.97 (14.00)	22.64 (14.02)	25.15 (14.05)	28.10 (15.16)	<0.001
**DRECE II** **1996**	**North-West**	**North**	**North-East**	**West**	**Central-South**	**East**	**South**	**Canary Islands**	
***n***	78	124	88	83	162	123	223	47	
**Age (years)**	48.29 (13.75)	46.03 (14.45)	48.95 (13.64)	46.05 (15.11)	47.92 (14.95)	45.65 (15.35)	45.53 (14.93)	44.26 (15.88)	0.248
**Sex (male)**	51 (65.38%)	73 (58.87%)	54 (61.36%)	51 (61.45%)	97 (59.88%)	84 (68.29%)	130 (58.30%)	26 (55.32%)	0.415
**BMI (kg/m^2^)**	27.79 (3.76)	26.69 (4.03)	28.61 (3.70)	26.08 (4.06)	26.86 (4.68)	27.21 (4.14)	28.36 (5.49)	27.75 (6.02)	<0.001
**Total energy intake (kcal/day)**	2474.43 (1061)	2212.04 (751.12)	2576.62 (2016)	2359.88 (769.19)	2178.05 (862.04)	2625.60 (1149)	2441.92 (1459)	1919.39 (770.33)	<0.001
**NOVA classification**									
**GROUP 4 (% of energy)**	25.91 (15.78)	28.87 (15.90)	25.53 (16.11)	25.06 (17.82)	26.95 (17.41)	21.85 (15.71)	23.13 (15.28)	29.33 (15.12)	0.010
**DRECE III** **2004**	**North-West**	**North**	**North-East**	**West**	**Central-South**	**East**	**South**	**Canary Islands**	
***n***	89	135	192	104	178	45	257	65	
**Age (years)**	44.18 (15.66)	44.68 (15.13)	47.80 (16.14)	44.96 (16.01)	45.29 (17.18)	44.93 (14.66)	44.17 (14.05)	51.17 (15.40)	0.061
**Sex (male)**	42 (47.19%)	64 (47.41%)	86 (44.79%)	46 (44.23%)	72 (40.45%)	21 (46.67%)	116 (45.14%)	28 (43.08%)	0.954
**BMI (kg/m^2^)**	28.52 (5.02)	26.59 (4.72)	27.80 (5.36)	26.70 (4.74)	26.34 (4.59)	28.23 (5.11)	28.38 (5.85)	28.40 (4.91)	<0.001
**Total energy intake (kcal/day)**	2286.15 (1080)	2580.85 (1353)	2485.81 (1109)	2408.26 (969.91)	2518.23 (1254)	2368.07 (771.36)	2114.92 (756.77)	2311.56 (906.83)	0.002
**NOVA classification**									
**GROUP 4 (% of energy)**	34.13 (18.02)	35.34 (15.59)	32.48 (12.55)	34.67 (14.78)	29.99 (19.68)	25.71 (11.57)	25.85 (13.51)	34.42 (12.85)	<0.001
**DRECE IV** **2008**	**North-West**	**North**	**North-East**	**West**	**Central-South**	**East**	**South**	**Canary Islands**	
***n***	562	370	833	373	1037	548	922	190	
**Age (years)**	44.06 (14.91)	45.58 (15.13)	43.51 (14.32)	43.28 (14.45)	43.81 (14.70)	44.58 (15.32)	42.88 (14.21)	42.14 (14.25)	0.067
**Sex (male)**	251 (44.66%)	176 (47.57%)	404 (48.50%)	180 (48.26%)	469 (45.23%)	271 (49.45%)	411 (44.58%)	95 (50.00%)	0.372
**BMI (kg/m^2^)**	26.26 (4.13)	24.67 (3.82)	25.30 (4.23)	26.88 (3.91)	25.17 (4.13)	24.84 (3.96)	26.60 (4.46)	25.87 (4.54)	0.007
**Total energy intake (kcal/day)**	2400.25 (910.08)	2382.53 (959.17)	2386.65 (985.88)	2490.63 (855.33)	2453.17 (860.99)	2432.02 (1021)	2491.67 (1003)	2530.22 (1001)	0.005
**NOVA classification**									
**GROUP 4 (% of energy)**	35.47 (16.94)	36.02 (18.33)	32.75 (19.76)	34.01 (17.48)	30.17 (17.14)	31.03 (17.60)	31.28 (18.33)	34.80 (16.12)	<0.001

Data is shown as mean (SD) or *n* (%).

## Data Availability

Additional data are available from the corresponding author on reasonable request.

## Supplementary Materials

The following supporting information can be downloaded at: https://www.mdpi.com/article/10.3390/nu14153223/s1. Table S1: Classification of items of the food frequency questionnaires according to degree of processing (NOVA classification). Table S2: Demographic characteristics of the DRECE cohorts.

## References

[1] Mendis S., Davis S., Norrving B. (2015). Organizational Update: The World Health Organization Global Status Report on Noncommunicable Diseases 2014; One More Landmark Step in the Combat against Stroke and Vascular Disease. Stroke.

[2] United Nations Political Declaration of the High-Level Meeting of the General Assembly on the Prevention and Control of Non-Communicable Diseases 2011. https://digitallibrary.un.org/record/710899/.

[3] Cena H., Calder P.C. (2020). Defining a Healthy Diet: Evidence for the Role of Contemporary Dietary Patterns in Health and Disease. Nutrients.

[4] Hawkes C. (2012). Food Policies for Healthy Populations and Healthy Economies. BMJ.

[5] Monteiro C.A. (2009). Nutrition and Health. The Issue Is Not Food, nor Nutrients, so Much as Processing. Public Health Nutr..

[6] World Health Organization (2003). Diet, Nutrition, and the Prevention of Chronic Diseases: Report of a WHO-FAO Expert Consultation.

[7] Wiseman M., Cannon G., Butrum R., Martin G., Higginbotham S., Jones C., Fletcher M. (2009). Policy and Action for Cancer Prevention. Food, Nutrition and Physical Activity: A Global Perspective.

[8] Stuckler D., McKee M., Ebrahim S., Basu S. (2012). Manufacturing Epidemics: The Role of Global Producers in Increased Consumption of Unhealthy Commodities Including Processed Foods, Alcohol, and Tobacco. PLoS Med..

[9] Monteiro C.A., Cannon G., Levy R., Moubarac J.-C., Jaime P., Martins A.P., Canella D., Louzada M., Parra D. (2016). NOVA. The Star Shines Bright. World Nutr..

[10] Monteiro C.A., Cannon G., Levy R.B., Moubarac J.-C., Louzada M.L., Rauber F., Khandpur N., Cediel G., Neri D., Martinez-Steele E. (2019). Ultra-Processed Foods: What They Are and How to Identify Them. Public Health Nutr..

[11] Moubarac J.-C., Batal M., Louzada M.L., Martinez Steele E., Monteiro C.A. (2017). Consumption of Ultra-Processed Foods Predicts Diet Quality in Canada. Appetite.

[12] Martínez Steele E., Popkin B.M., Swinburn B., Monteiro C.A. (2017). The Share of Ultra-Processed Foods and the Overall Nutritional Quality of Diets in the US: Evidence from a Nationally Representative Cross-Sectional Study. Popul. Health Metr..

[13] da Costa Louzada M.L., Ricardo C.Z., Steele E.M., Levy R.B., Cannon G., Monteiro C.A. (2018). The Share of Ultra-Processed Foods Determines the Overall Nutritional Quality of Diets in Brazil. Public Health Nutr..

[14] Marrón-Ponce J.A., Flores M., Cediel G., Monteiro C.A., Batis C. (2019). Associations between Consumption of Ultra-Processed Foods and Intake of Nutrients Related to Chronic Non-Communicable Diseases in Mexico. J. Acad. Nutr. Diet..

[15] Parra D.C., da Costa-Louzada M.L., Moubarac J.-C., Bertazzi-Levy R., Khandpur N., Cediel G., Monteiro C.A. (2019). Association between Ultra-Processed Food Consumption and the Nutrient Profile of the Colombian Diet in 2005. Salud Publica Mex..

[16] Elizabeth L., Machado P., Zinöcker M., Baker P., Lawrence M. (2020). Ultra-Processed Foods and Health Outcomes: A Narrative Review. Nutrients.

[17] Kelly B., Jacoby E. (2018). Public Health Nutrition Special Issue on Ultra-Processed Foods. Public Health Nutr..

[18] Casas R. (2022). Moving towards a Healthier Dietary Pattern Free of Ultra-Processed Foods. Nutrients.

[19] Martínez Steele E., Baraldi L.G., da Costa Louzada M.L., Moubarac J.-C., Mozaffarian D., Monteiro C.A. (2016). Ultra-Processed Foods and Added Sugars in the US Diet: Evidence from a Nationally Representative Cross-Sectional Study. BMJ Open.

[20] Rauber F., da Costa Louzada M.L., Steele E.M., Millett C., Monteiro C.A., Levy R.B. (2018). Ultra-Processed Food Consumption and Chronic Non-Communicable Diseases-Related Dietary Nutrient Profile in the UK (2008–2014). Nutrients.

[21] Moodie R., Stuckler D., Monteiro C., Sheron N., Neal B., Thamarangsi T., Lincoln P., Casswell S., Lancet NCD Action Group (2013). Profits and Pandemics: Prevention of Harmful Effects of Tobacco, Alcohol, and Ultra-Processed Food and Drink Industries. Lancet.

[22] Moubarac J.-C., Martins A.P.B., Claro R.M., Levy R.B., Cannon G., Monteiro C.A. (2013). Consumption of Ultra-Processed Foods and Likely Impact on Human Health. Evidence from Canada. Public Health Nutr..

[23] Adams J., White M. (2015). Characterisation of UK Diets According to Degree of Food Processing and Associations with Socio-Demographics and Obesity: Cross-Sectional Analysis of UK National Diet and Nutrition Survey (2008–12). Int. J. Behav. Nutr. Phys. Act..

[24] Batal M., Johnson-Down L., Moubarac J.-C., Ing A., Fediuk K., Sadik T., Tikhonov C., Chan L., Willows N. (2018). Quantifying Associations of the Dietary Share of Ultra-Processed Foods with Overall Diet Quality in First Nations Peoples in the Canadian Provinces of British Columbia, Alberta, Manitoba and Ontario. Public Health Nutr..

[25] Bielemann R.M., Motta J.V.S., Minten G.C., Horta B.L., Gigante D.P. (2015). Consumption of Ultra-Processed Foods and Their Impact on the Diet of Young Adults. Rev. Saude Publica.

[26] Cediel G., Reyes M., da Costa Louzada M.L., Martinez Steele E., Monteiro C.A., Corvalán C., Uauy R. (2018). Ultra-Processed Foods and Added Sugars in the Chilean Diet (2010). Public Health Nutr..

[27] Cornwell B., Villamor E., Mora-Plazas M., Marin C., Monteiro C.A., Baylin A. (2018). Processed and Ultra-Processed Foods Are Associated with Lower-Quality Nutrient Profiles in Children from Colombia. Public Health Nutr..

[28] Vandevijvere S., De Ridder K., Fiolet T., Bel S., Tafforeau J. (2019). Consumption of Ultra-Processed Food Products and Diet Quality among Children, Adolescents and Adults in Belgium. Eur. J. Nutr..

[29] Juul F., Martinez-Steele E., Parekh N., Monteiro C.A., Chang V.W. (2018). Ultra-Processed Food Consumption and Excess Weight among US Adults. Br. J. Nutr..

[30] Nardocci M., Leclerc B.-S., Louzada M.-L., Monteiro C.A., Batal M., Moubarac J.-C. (2019). Consumption of Ultra-Processed Foods and Obesity in Canada. Can. J. Public Health.

[31] Julia C., Martinez L., Allès B., Touvier M., Hercberg S., Méjean C., Kesse-Guyot E. (2018). Contribution of Ultra-Processed Foods in the Diet of Adults from the French NutriNet-Santé Study. Public Health Nutr..

[32] da Costa Louzada M.L., Baraldi L.G., Steele E.M., Martins A.P.B., Canella D.S., Moubarac J.-C., Levy R.B., Cannon G., Afshin A., Imamura F. (2015). Consumption of Ultra-Processed Foods and Obesity in Brazilian Adolescents and Adults. Prev. Med..

[33] Canella D.S., Levy R.B., Martins A.P.B., Claro R.M., Moubarac J.-C., Baraldi L.G., Cannon G., Monteiro C.A. (2014). Ultra-Processed Food Products and Obesity in Brazilian Households (2008–2009). PLoS ONE.

[34] Pan American Health Organization Ultra-Processed Food and Drink Products in Latin America: Trends, Impact on Obesity, Policy Implications. https://iris.paho.org/handle/10665.2/7699.

[35] Neri D., Steele E.M., Khandpur N., Cediel G., Zapata M.E., Rauber F., Marrón-Ponce J.A., Machado P., da Costa Louzada M.L., Andrade G.C. (2022). Ultraprocessed Food Consumption and Dietary Nutrient Profiles Associated with Obesity: A Multicountry Study of Children and Adolescents. Obes. Rev..

[36] Monteiro C.A., Moubarac J.C., Levy R.B., Canella D.S., da Costa Louzada M.L., Cannon G. (2018). Household Availability of Ultra-Processed Foods and Obesity in Nineteen European Countries. Public Health Nutr..

[37] de Deus Mendonça R., Lopes A.C.S., Pimenta A.M., Gea A., Martinez-Gonzalez M.A., Bes-Rastrollo M. (2017). Ultra-Processed Food Consumption and the Incidence of Hypertension in a Mediterranean Cohort: The Seguimiento Universidad de Navarra Project. Am. J. Hypertens..

[38] Martinez-Perez C., San-Cristobal R., Guallar-Castillon P., Martínez-González M.Á., Salas-Salvadó J., Corella D., Castañer O., Martinez J.A., Alonso-Gómez Á.M., Wärnberg J. (2021). Use of Different Food Classification Systems to Assess the Association between Ultra-Processed Food Consumption and Cardiometabolic Health in an Elderly Population with Metabolic Syndrome (PREDIMED-Plus Cohort). Nutrients.

[39] Fiolet T., Srour B., Sellem L., Kesse-Guyot E., Allès B., Méjean C., Deschasaux M., Fassier P., Latino-Martel P., Beslay M. (2018). Consumption of Ultra-Processed Foods and Cancer Risk: Results from NutriNet-Santé Prospective Cohort. BMJ.

[40] Kim H., Hu E.A., Rebholz C.M. (2019). Ultra-Processed Food Intake and Mortality in the USA: Results from the Third National Health and Nutrition Examination Survey (NHANES III, 1988–1994). Public Health Nutr..

[41] Bonaccio M., Di Castelnuovo A., Costanzo S., De Curtis A., Persichillo M., Sofi F., Cerletti C., Donati M.B., de Gaetano G., Iacoviello L. (2021). Ultra-Processed Food Consumption Is Associated with Increased Risk of All-Cause and Cardiovascular Mortality in the Moli-Sani Study. Am. J. Clin. Nutr..

[42] Rico-Campà A., Martínez-González M.A., Alvarez-Alvarez I., de Deus Mendonça R., de la Fuente-Arrillaga C., Gómez-Donoso C., Bes-Rastrollo M. (2019). Association between Consumption of Ultra-Processed Foods and All Cause Mortality: SUN Prospective Cohort Study. BMJ.

[43] Blanco-Rojo R., Sandoval-Insausti H., López-Garcia E., Graciani A., Ordovás J.M., Banegas J.R., Rodríguez-Artalejo F., Guallar-Castillón P. (2019). Consumption of Ultra-Processed Foods and Mortality: A National Prospective Cohort in Spain. Mayo Clin. Proc..

[44] Romero Ferreiro C., Martín-Arriscado Arroba C., Cancelas Navia P., Lora Pablos D., Gómez de la Cámara A. (2021). Ultra-Processed Food Intake and All-Cause Mortality: DRECE Cohort Study. Public Health Nutr..

[45] Imamura F., Micha R., Khatibzadeh S., Fahimi S., Shi P., Powles J., Mozaffarian D. (2015). Dietary Quality among Men and Women in 187 Countries in 1990 and 2010: A Systematic Assessment. Lancet Glob. Health.

[46] Monteiro C.A., Moubarac J.-C., Cannon G., Ng S.W., Popkin B. (2013). Ultra-Processed Products Are Becoming Dominant in the Global Food System. Obes Rev..

[47] Latasa P., da Coasta Louzada M.L., Martinez Steele E., Monteiro C.A. (2018). Added Sugars and Ultra-Processed Foods in Spanish Households (1990–2010). Eur. J. Clin. Nutr..

[48] Haro J.M., Tyrovolas S., Garin N., Diaz-Torne C., Carmona L., Sanchez-Riera L., Perez-Ruiz F., Murray C.J. (2014). The Burden of Disease in Spain: Results from the Global Burden of Disease Study 2010. BMC Med..

[49] Shojaei E., Rexachs D., Wong A., Epelde F., Luque E. (2020). A Method for Projections of the Emergency Department Behaviour by Non-Communicable Diseases From 2019 to 2039. IEEE J. Biomed. Health Inform..

[50] Trichopoulou A., Naska A., Costacou T., DAFNE III Group (2002). Disparities in Food Habits across Europe. Proc. Nutr. Soc..

[51] Gómez-Jerique J.A., Rubio Herrera M.A., Gómez De La Cámara A., Gutiérrez Fuentes J.A. (2011). Diet and Cardiovascular Risk in Spain Study (DRECE) Capítulo 2. El proyecto DRECE. Med. Clin..

[52] Martin-Moreno J.M., Boyle P., Gorgojo L., Maisonneuve P., Fernandez-Rodriguez J.C., Salvini S., Willett W.C. (1993). Development and Validation of a Food Frequency Questionnaire in Spain. Int. J. Epidemiol..

[53] Rodríguez I.T., Ballart J.F., Pastor G.C., Jordà E.B., Val V.A. (2008). Validation of a short questionnaire on frequency of dietary intake: Reproducibility and validity. Nutr. Hosp..

[54] Panel de Consumo Alimentario. https://www.mapa.gob.es/es/alimentacion/temas/consumo-tendencias/panel-de-consumo-alimentario/.

[55] Monteiro C.A., Cannon G., Moubarac J.-C., Levy R.B., Louzada M.L.C., Jaime P.C. (2018). The UN Decade of Nutrition, the NOVA Food Classification and the Trouble with Ultra-Processing. Public Health Nutr..

[56] Greenacre M.J. (2008). La Práctica del Análisis de Correspondencias.

[57] Romero Ferreiro C., Lora Pablos D., Gómez de la Cámara A. (2021). Two Dimensions of Nutritional Value: Nutri-Score and NOVA. Nutrients.

[58] Hedeker D., Gibbons R.D. (2006). Longitudinal Data Analysis.

[59] Willett W.C., Sacks F., Trichopoulou A., Drescher G., Ferro-Luzzi A., Helsing E., Trichopoulos D. (1995). Mediterranean Diet Pyramid: A Cultural Model for Healthy Eating. Am. J. Clin. Nutr..

[60] Bonaccio M., Costanzo S., Di Castelnuovo A., Persichillo M., Magnacca S., De Curtis A., Cerletti C., Donati M.B., de Gaetano G., Iacoviello L. (2022). Ultra-Processed Food Intake and All-Cause and Cause-Specific Mortality in Individuals with Cardiovascular Disease: The Moli-Sani Study. Eur. Heart J..

[61] Juul F., Hemmingsson E. (2015). Trends in Consumption of Ultra-Processed Foods and Obesity in Sweden between 1960 and 2010. Public Health Nutr..

[62] Wang L., Martínez Steele E., Du M., Pomeranz J.L., O’Connor L.E., Herrick K.A., Luo H., Zhang X., Mozaffarian D., Zhang F.F. (2021). Trends in Consumption of Ultraprocessed Foods Among US Youths Aged 2–19 Years, 1999–2018. JAMA.

[63] de Araújo T.P., de Moraes M.M., Magalhães V., Afonso C., Santos C., Rodrigues S.S.P. (2021). Ultra-Processed Food Availability and Noncommunicable Diseases: A Systematic Review. Int. J. Environ. Res. Public Health.

[64] Boyland E.J., Nolan S., Kelly B., Tudur-Smith C., Jones A., Halford J.C., Robinson E. (2016). Advertising as a Cue to Consume: A Systematic Review and Meta-Analysis of the Effects of Acute Exposure to Unhealthy Food and Nonalcoholic Beverage Advertising on Intake in Children and Adults. Am. J. Clin. Nutr.

[65] Marti A. (2019). Ultra-Processed Foods Are Not “Real Food” but Really Affect Your Health. Nutrients.

[66] Oxandabarat A. OPS/OMS Uruguay—Guía Alimentaria para la Población Uruguaya | OPS/OMS. https://www3.paho.org/uru/index.php?option=com_content&view=article&id=1375:guia-alimentaria-para-la-poblacion-uruguaya&Itemid=451.

[67] Monteiro C.A., Cannon G., Moubarac J.-C., Martins A.P.B., Martins C.A., Garzillo J., Canella D.S., Baraldi L.G., Barciotte M., Louzada M.L. (2015). da C.; et al. Dietary Guidelines to Nourish Humanity and the Planet in the Twenty-First Century. A Blueprint from Brazil. Public Health Nutr..

[68] Mauricio H.-F., Batis C., Rivera J.A., Colchero M.A. (2019). Reduction in Purchases of Energy-Dense Nutrient-Poor Foods in Mexico Associated with the Introduction of a Tax in 2014. Prev. Med..

[69] Bíró A. (2015). Did the Junk Food Tax Make the Hungarians Eat Healthier?. Food Policy.

[70] Baraldi L.G., Martinez Steele E., Canella D.S., Monteiro C.A. (2018). Consumption of Ultra-Processed Foods and Associated Sociodemographic Factors in the USA between 2007 and 2012: Evidence from a Nationally Representative Cross-Sectional Study. BMJ Open.

[71] Khandpur N., Cediel G., Obando D.A., Jaime P.C., Parra D.C. (2020). Factores sociodemográficos asociados al consumo de alimentos ultraprocesados en Colombia. Rev. Saúde Pública.

[72] de Deus Mendonça R., Pimenta A.M., Gea A., de la Fuente-Arrillaga C., Martinez-Gonzalez M.A., Lopes A.C.S., Bes-Rastrollo M. (2016). Ultraprocessed Food Consumption and Risk of Overweight and Obesity: The University of Navarra Follow-Up (SUN) Cohort Study. Am. J. Clin. Nutr..

[73] Beslay M., Srour B., Méjean C., Allès B., Fiolet T., Debras C., Chazelas E., Deschasaux M., Wendeu-Foyet M.G., Hercberg S. (2020). Ultra-Processed Food Intake in Association with BMI Change and Risk of Overweight and Obesity: A Prospective Analysis of the French NutriNet-Santé Cohort. PLoS Med..

[74] Pestoni G., Habib L., Reber E., Rohrmann S., Staub K., Stanga Z., Faeh D. (2021). Ultraprocessed Food Consumption Is Strongly and Dose-Dependently Associated with Excess Body Weight in Swiss Women. Obesity.

[75] Chandon P., Wansink B. (2012). Does Food Marketing Need to Make Us Fat? A Review and Solutions. Nutr. Rev..

[76] Gómez de la Cámara A., De Andrés Esteban E., Urrútia Cuchí G., Calderón Sandubete E., Rubio Herrera M.Á., Menéndez Orenga M., Lora Pablos D. (2017). Variability of Nutrients Intake, Lipid Profile and Cardiovascular Mortality among Geographical Areas in Spain: The DRECE Study. Geospat. Health.

[77] Moreiras-Varela O. (1989). The Mediterranean Diet in Spain. Eur. J. Clin. Nutr..

[78] Dinu M., Tristan Asensi M., Pagliai G., Lotti S., Martini D., Colombini B., Sofi F. (2022). Consumption of Ultra-Processed Foods Is Inversely Associated with Adherence to the Mediterranean Diet: A Cross-Sectional Study. Nutrients.

[79] Instituto Nacional de Estadística (National Statistics Institute). https://www.ine.es/dynt3/inebase/index.htm?padre=1928&capsel=1928.

