# Supplementary material for: Geographical and Temporal Variability of Ultra-Processed Food Consumption in the Spanish Population: Findings from the DRECE Study
Source: Nutrients. 2022 Aug 6;14(15):3223. doi: 10.3390/nu14153223 (PMC9370340; doi:10.3390/nu14153223)
Supplement: Supplementary file 1 [file nutrients-14-03223-s001.zip › nutrients-1839204-supplementary.pdf]

Table S1: Classification of items of the food frequency questionnaires according to degree of processing (NOVA classification)

NOVA GROUPS
**Unprocessed or minimally processed foods (Group 1)**<br>Fruit, vegetables, legumes, eggs, meats, poultry, fish and seafood, nuts, plain yogurt, milk (whole, semi-skimmed, and non-fat), natural juice, coffee and tea
**Processed culinary ingredients (Group 2)**<br>Sugar, honey, jam, vegetable oils (olive, sunflower) and butter
**Processed foods (Group 3)**<br>Cheeses, cured traditional ham, canned fish, breads (white and whole), olives, beer and wine
**Ultra-processed foods (Group 4)**<br>Ham, processed meat (cold cuts, sausage, hamburger), pate, foie-gras, ultra-processed cheese, salty snacks, pizza, sauces and dressings, liquors, sugar sweetened beverages, low calorie sugar sweetened beverages, juice boxes, milkshakes, cookies, chocolate cookies, muffins, doughnuts, cakes or other non-handmade pastries, churros, chocolates and candies, breakfast cereals, sweetened yoghurt, sugared 'fruit' yoghurts, Petit Suisse, dairy desserts, ice cream and margarine

Table S2: Demographic characteristics of the DRECE cohorts

	N	N included	Age (years)	Sex (male)	BMI (kg/m2)
**DRECE I 1991**	4787	4679	30.56 (15.61) [5-60]	2288 (48.90%)	24.24 (5.04)
**DRECE II 1996**	1079	928	46.56 (14.78) [10-73]	566 (60.99%)	27.47 (4.68)
**DRECE III 2004**	2009	1065	45.84 (15.99) [5-73]	475 (44.60%)	27.61 (5.24)
**DRECE IV 2008**	5038	4835	43.73 (14.65) [15-74]	2257 (46.68%)	26.22 (4.18)

Data is shown as mean (SD), [min.-max.] or N (%)
